# Antithyroglobulin Antibody Variation During Follow-Up Has a Good Prognostic Value for Preoperative Antithyroglobulin Antibody-Positive Differentiated Thyroid Cancer Patients: A Retrospective Study in Southwest China

**DOI:** 10.3389/fendo.2021.774275

**Published:** 2021-12-15

**Authors:** Qianhui Liu, Mengting Yin, Guixing Li

**Affiliations:** Department of Laboratory Medicine, West China Hospital of Sichuan University, Chengdu, China

**Keywords:** thyroid cancer, prognosis, antithyroglobulin, biomarker, DTC (differentiated thyroid cancer)

## Abstract

**Objective:**

Antithyroglobulin antibody (TgAb) is a potential tumour marker for detecting differentiated thyroid cancer (DTC) recurrence, but insufficient data have supported its clinical applications. Our study aimed to describe the changing trend of TgAb after surgery and identify the relationship between this trend and clinical outcomes.

**Patients and Methods:**

We reviewed the electronic records of 1,686 DTC patients who had undergone total thyroidectomy (TT) and radioactive iodine (^131^I) therapy at West China Hospital of Sichuan University from January 2015 to December 2017. Finally, 289 preoperative TgAb-positive DTC patients were included and divided into four subgroups depending on the clinical outcome: Group A (tumour free), Group B (uncertain), Group C (incomplete biochemical response), and Group D (structural disease). The patient demographics, tumour characteristics, operations, pathology reports, and all serological biomarkers were reviewed and compared, and the prognostic efficacy of TgAb was evaluated.

**Results:**

Among all 1,686 patients, 393 (23.65%) were TgAb positive (>40 IU/ml) preoperatively. The TgAb level in Group A decreased significantly after surgery and ^131^I therapy and stabilised at a low level after 1–2 years of ^131^I therapy. However, in the other three groups, the decrease in TgAb was not significant after treatment. Conversely, TgAb declined slowly and remained stable or increased. The variations in TgAb relative to the preoperative level of Group A were significantly larger than those of Groups B, C, and D at most time points of follow-up (p < 0.001). By receiver operating characteristic (ROC) analyses, the variations of TgAb > −77.9% at 6 months after ^131^I therapy (area under the curve (AUC) = 0.862; p < 0.001) and TgAb > −88.6% at 2 years after ^131^I therapy (AUC = 0.901; p < 0.001) had good prognostic efficacy in tumour-free survival. When the variation in TgAb > −88.6% at 2 years after ^131^I therapy was incorporated as a variable in the American Thyroid Association (ATA) categories, both intermediate- and high-risk patients also had a significantly increased chance of being tumour free (from 75.68% to 93.88% and 42.0% to 82.61%, respectively).

**Conclusions:**

For preoperative TgAb-positive DTC patients, variations in TgAb > −77.9% at 6 months after ^131^I therapy and TgAb > −88.6% at 2 years after ^131^I therapy had good prognostic efficacy. Their incorporation as variables in the ATA risk stratification system could more accurately predict disease-free survival.

## Introduction

Thyroid cancer is currently the fifth most common cancer diagnosis in women. By 2030, it is estimated to be the second leading cancer diagnosis in women and the ninth leading cancer diagnosis in men (1). Differentiated thyroid cancer (DTC) accounts for most thyroid cancers, mainly papillary thyroid cancer (PTC) and follicular thyroid cancer (FTC). The prognosis of DTC patients is good; however, instances of persistent or recurrent disease, such as local lymph node metastasis, are not uncommon; thus, long-term observation after the surgery of DTC patients is necessary. In the follow-up of DTC patients, thyroglobulin (Tg) is the most sensitive and specific tumour marker for the early detection of recurrence (2). However, antithyroglobulin antibody (TgAb) shows potential interference with the Tg assay, particularly the immunometric assay (IMA), by forming the Tg–TgAb complex, compromising the clinical usefulness of monitoring Tg in DTC patients for recurrence (3, 4). Thus, simultaneous measurement of TgAb is essential (5).

In the follow-up of DTC, the greatest challenge is patients with serum Tg–IMA concentrations suggesting tumour absence in whom it is not possible to ensure whether this finding indicates complete remission or underestimated Tg due to the interference of TgAb (6). Many studies have focused on improving Tg detection technologies, such as liquid chromatography/tandem mass spectrometry and radioimmunoassay, but the former may cause false-negative results in many patients with structural disease (7–9); the latter is unsuitable for large-scale clinical applications and may cause false-positive results positives.

Currently, Tg measurement is widely performed using second-generation IMA in practical clinical work, and interference with detectable TgAb is inevitable. Currently, no optimal assay exists to detect Tg that can avoid interference from TgAb. Theoretically, the body produces TgAb because of Tg expression; thus, TgAb concentrations should respond to changes in Tg-secreting thyroid tissue. For DTC patients who have undergone total thyroidectomy (TT) and ^131^I therapy, no residual thyroid tissue should be detected in the body theoretically; ideally, TgAb should be undetectable. Therefore, we are justified in holding that the TgAb trend can be used as a more reliable tumour marker in the follow-up of DTC patients and can be a better predictor of persistent/recurrent disease.

Although some studies have focused on the relationship between TgAb and the clinical outcomes of DTC, the current literature does not provide sufficient data to provide evidence-based answers to many questions arising in the care of TgAb-positive DTC patients (10). The general conclusion is that a reduction >50% in the TgAb concentration is associated with a low risk of persistent/recurrent disease (11, 12). However, a 50% TgAb decline was established empirically, not statistically. Our study calculated variations in TgAb at every follow-up time point for each subject, describing the long-term dynamic change trend of TgAb and finding the relationship between this trend and the clinical outcome.

## Materials and Methods

### Study Objectives

This was a retrospective study approved by the Ethics Committee of West China Hospital of Sichuan University. We reviewed the electronic records of 1,686 DTC patients who had undergone TT and radioactive iodine (^131^I) therapy at our hospital from January 2015 to December 2017. The patient demographics, tumour characteristics, operations, pathology reports, and all serological biomarkers were carefully reviewed. The tumours were grouped into stages according to the 8th Tumour-Node-Metastasis Classification of the American Joint Committee on Cancer, and the risk of recurrence category followed the 2015 American Thyroid Association (ATA) risk stratification system (2).

In this study, all operations were performed by three experienced thyroid surgeons who had more than 10 years of experience in thyroid operations in West China Hospital. All the patients had undergone TT, and neck lymph node dissections were performed according to preoperative imaging studies, the levels of serological indicators, and intraoperative morphological appearance. After surgery, all the study patients had received ablative ^131^I therapy. Treatment dosing was determined empirically using 30 to 100 mCi for thyroid remnant ablation and 100 to 200 mCi for postoperative residual neck lymph node metastases.

### Follow-Up

The thyroid-stimulating hormone (TSH), FT3, FT4, Tg, and TgAb levels were measured 1 month after surgery, before ablative ^131^I therapy, and 1 and 6 months after ablative ^131^I therapy. Next, the patients were followed up by annual measurements of the TSH, FT3, FT4, Tg, and TgAb levels and neck ultrasound. Other imaging methods, such as chest and mediastinal CT and fluorine-18 fluorodeoxyglucose–positron emission tomography (FDG–PET)/CT, were performed when the basal Tg level (with suppressed T4 and TgAb negativity) >1 ng/ml or the TgAb level remained positive or was increasing. For patients with persistent/recurrent disease, additional ^131^I therapy and repeated surgery were performed at the discretion of the attending physician.

### Definitions of Clinical Outcomes

All the clinical data, including imaging findings (neck ultrasound in all patients, diagnostic whole-body ^131^I scintigraphy, and chest and mediastinal CT, and FDG-PET/CT in selected patients), the basal Tg levels, TgAb levels, fine-needle aspiration (FNA), and postoperative pathological reports, were used to define the clinical outcomes.

Patients were considered tumour free if they had suppressed Tg < 1 ng/ml, TgAb < 40 IU/ml, and no structural evidence of disease. Patients were considered to have persistent disease if they had suppressed Tg ≥ 1 ng/ml or TgAb ≥ 40 IU/ml with a continuous increase, any evidence of disease on imaging, or biopsy-proven disease (cytology or histology). Patients with persistent disease were further classified as having an incomplete biochemical response (suppressed Tg > 1 ng/ml or TgAb ≥ 40 IU/ml with a continuous increase without a structural correlate). Patients were considered to have structural disease if cytology/histology was positive or imaging findings were highly suspicious for metastatic disease. Patients with suppressed Tg < 1 ng/ml, TgAb ≥ 40 IU/ml, and stability during the entire follow-up were considered uncertain.

### Laboratory Analysis

The high-sensitivity electrochemiluminescence immunoassay (Roche Diagnostics GmbH; cobas e 601 from 2015 to 2020 and cobas e 801 from 2020) was used to measure TSH (measuring range: 0.005–100 μIU/ml), FT3 (0.6–50 pmol/L), FT4 (0.5–100 pmol/L), Tg (0.1–1,000 ng/ml from 2015–2016 and 0.04–500 ng/ml from 2016), and TgAb (10–4,000 IU/ml). All the TSH, FT3, FT4, Tg, and TgAb levels were measured at the same laboratory in our hospital. Sera showing TgAb levels >40 IU/ml were considered positive for DTC patients (13).

### Statistical Analysis

All the continuous variables were tested for a normal distribution using the Kolmogorov–Smirnov (K-S) normality test. Next, the normally distributed variables were expressed as means ± standard deviation (M ± SD), and skewed variables were expressed as medians (minimum value, maximum value). The differences between the two groups were examined using unpaired two-tailed Student’s t-test and the Mann–Whitney U test for normally and non-normally distributed parameters, respectively. Categorical variables were compared by chi-squared test. Receiver operating characteristic (ROC) curves were used to evaluate the predictive effect of TgAb on the clinical outcome. Binary logistic regression was used to analyse the risk factors for the persistence/recurrence of disease. p-Values <0.05 were considered statistically significant. All statistical analyses were performed using SPSS 25.0.

## Results

### Characteristics of the Patients

**Figure 1:**
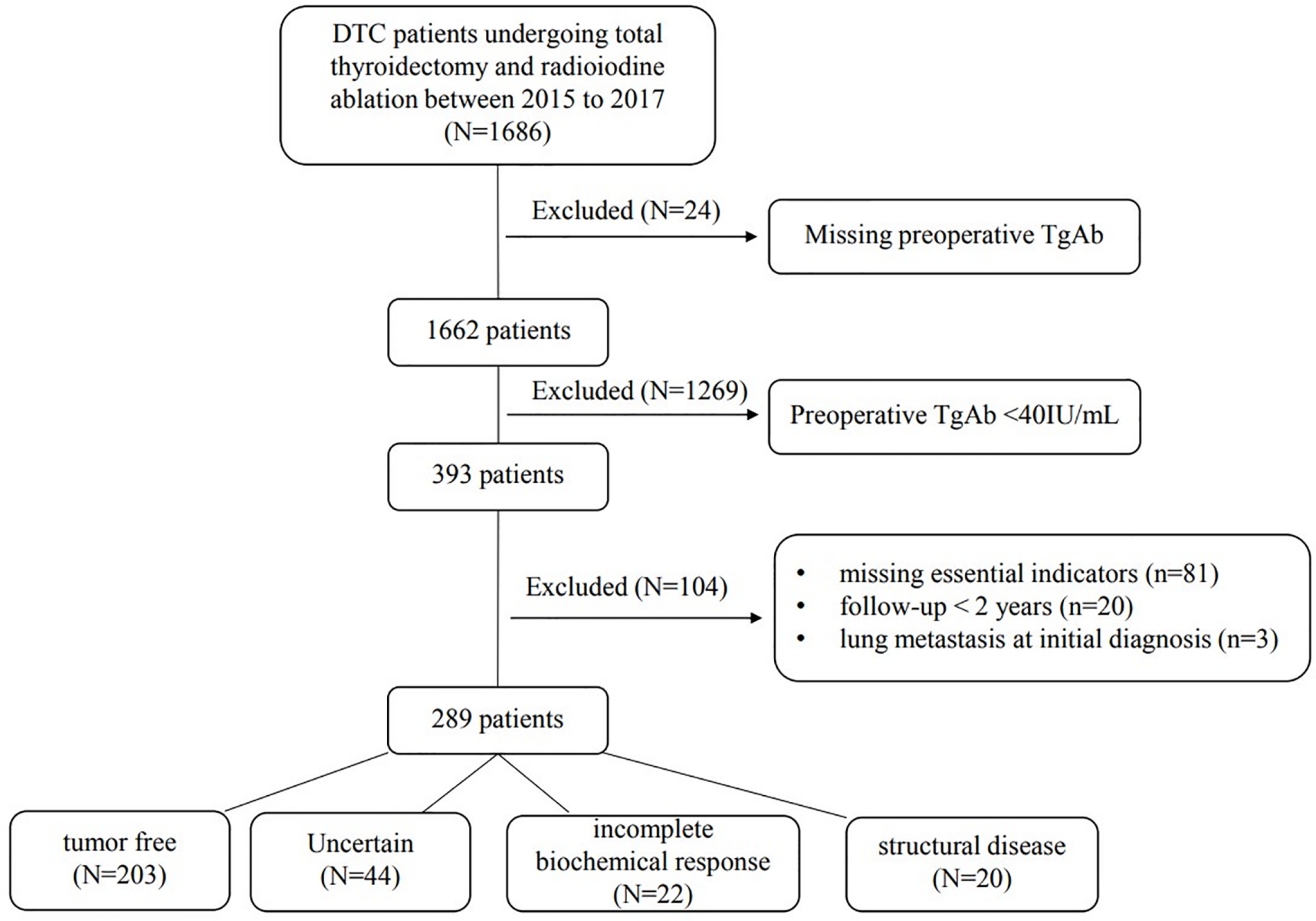
Flowchart of patient selection.

Of the 1,686 consecutive DTC patients managed at our hospital from January 2015 to December 2017, 24 were excluded because preoperative TgAb was missing. Among the 1,662 patients, 393 (23.65%) had a preoperative TgAb > 40 IU/ml. Subsequent exclusions comprised 81 cases in which the patients had missing essential indicators [missing TgAb in the follow-up (n = 52), missing essential items to define the outcome (n = 29)], 20 cases in which a follow-up period was less than 2 years and 3 cases with lung metastasis at the initial diagnosis. Thus, the final cohort comprised 289 patients (**Figure 1**).

Most of the patients were female (85.12%) and had classic PTC (96.54%). The median age at diagnosis was 39 years (range: 16–74 years). The median lesion diameter was 1.2 cm (range: 0.3–6.0 cm), and bilateral and multifocal lesions were present in 103 patients (35.64%) and 118 patients (40.83%), respectively. Extrathyroidal invasion was documented in 227 patients (78.55%), of whom 198 (68.52%) had capsular or fatty tissue invasion and 29 (10.03%) had invasion of nerves, muscles, blood vessels, and the windpipe. A total of 250 (86.51%) patients presented with lymph node involvement, with a median number of invaded lymph nodes of 4 (range: 0–36). The ATA risk stratification was classified as low in 17 patients (5.88%), intermediate in 222 patients (76.82%), and high in 50 patients (17.30%). The characteristics of the study cohort are indicated in **Table 1**.

**Table 1:** Characteristics of the study patients.

Characteristics	Value
No. of patients	289
Age at diagnosis (years), median (range)	39 (16–74)
<55	267 (92.39%)
≥55	22 (7.61%)
Sex	
Male	43 (14.88%)
Female	246 (85.12%)
Surgery	
TT + central neck lymph node dissection	189 (65.40%)
TT + central and lateral neck lymph node dissection	100 (34.60%)
Histology	
Classic PTC	279 (96.54%)
FV-PTC	5 (1.73%)
DSV-PTC	5 (1.73%)
Primary tumour size (cm), median (range)	1.2 (0.3–6.0)
No. of LN metastases, median (range)	4 (0–36)
Bilateral tumour	103 (35.64%)
Multifocal tumour	118 (40.83%)
Extrathyroidal extension	227 (78.55%)
Microscopic	198 (68.52%)
Macroscopic	29 (10.03%)
T	
T1	210 (72.66%)
T2	39 (13.49%)
T3	26 (9.00%)
T4	14 (4.84%)
N	
N0	39 (13.49%)
N1a	160 (55.36%)
N1b	90 (31.14%)
Staging	
<55 years	
I	267 (92.39%)
II	0 (0%)
≥55 years	
I	3 (1.04%)
II	14 (4.84%)
III	3 (1.04%)
IV	2 (0.69%)
Initial risk stratification	
Low risk	17 (5.88%)
Intermediate risk	222 (76.82%)
High risk	50 (17.30%)
Cumulative ^131^I-administered activities (mCi)	
30	20 (6.92%)
50	8 (2.77%)
100	244 (84.43%)
150	17 (5.88%)
Time interval between the first ^131^I treatments to surgery (months), median (range)	4.0 (0.8–12.0)
Preoperative serum biomarkers level	
TSH (μIU/ml), median (range)	3.05 (0.01–52.99)
FT3 (pmol/L), median (range)	4.82 (3.27–8.53)
FT4 (pmol/L), median (range)	16.31 (9.63–27.30)
TgAb (IU/ml), median (range)	374.40 (44.74–4,000)
Follow-up information	
Median follow-up (years), median (range)	4.83 (0.99–6.50)
Final status	
Tumour free	203 (70.24%)
Uncertain	44 (15.22%)
Incomplete biochemical response	22 (7.61%)
Structural disease	20 (6.92%)

TT, total thyroidectomy; PTC, papillary thyroid cancer; FV, follicular variant; DSV, diffuse sclerosing variant; LN, lymph node; TSH, thyroid-stimulating hormone; TgAb, antithyroglobulin antibody.

### Change Trends of Antithyroglobulin Antibody and Comparisons of the Levels of Antithyroglobulin Antibody at Different Time Points

**Figure 2:**
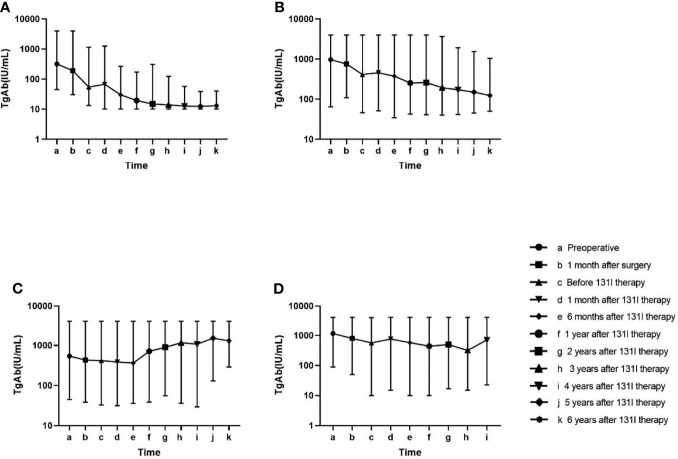
Change in the antithyroglobulin antibody (TgAb) level in the follow-up of four groups. Patients in Group **(A)** had lower TgAb concentrations than the other three groups at every time point in the follow-up (p < 0.05). Patients in Group **(B)** had lower TgAb concentrations than those in Group **(C)** at the late follow-up (p < 0.05). No significant difference was found in the TgAb values at other time points among Groups **(B–D)** (**Table 2**).

Change trends of TgAb in the four groups are displayed in **Figure 2**. In the tumour-free group (A), TgAb significantly decreased after surgery and ^131^I therapy. Approximately 1 year after ^131^I therapy (f), TgAb decreased slightly, and the concentration remained low. In the uncertain group (B), TgAb decreased after surgery and ^131^I therapy, but not significantly. In the follow-up, TgAb declined slowly and remained positive. In the incomplete biochemical response (C) and structural disease (D) groups, no significant decrease in TgAb was observed after treatment, and the TgAb concentrations remained high or increased during the follow-up. In all groups except Group C, small increases were observed in the levels of TgAb at 1 month after ^131^I therapy (d) compared with those before ^131^I therapy (c) (**Table 2**).

Table 2Levels of TgAb at every time point in the 4 groups.

**Table:** 

TgAb (IU/ml), median (range)	Tumour free (A)	Uncertain (B)	Incomplete biochemical response (C)	Structural disease (D)
Preoperative (a)	320.60 (44.92–4000)	970.80 (63.94–4000)	541.90 (44.74–4000)	1148.85 (88.74–4000)
1 month after surgery (b)	190.60 (30.44–4000)	754.25 (108.60–4000)	431.95 (38.27–4000)	791.80 (50.00–4000)
Before ^131^I therapy (c)	54.12 (13.28–1165)	412.6 (45.87–4000)	418.7 (32.91–4000)	563.25 (10.00–3916)
1 month after ^131^I therapy (d)	67.17 (10.00–1245)	454.75 (51.00–4000)	385.50 (31.24–4000)	757.70 (15.00–4000)
6 months after ^131^I therapy (e)	30.00 (10.00–267.00)	373.95 (34.48–4000)	364.30 (35.76–4000)	574.70 (10.00–4000)
1 year after ^131^I therapy (f)	19.48 (10.00–172.10)	254.05 (42.48–4000)	716 (38.59–4000)	437.40 (10.00–4000)
2 years after ^131^I therapy (g)	14.90 (10.00–307.70)	260.00 (40.74–4000)	900.35 (55.59–4000)	494.00 (16.81–4000)
3 years after ^131^I therapy (h)	13.93 (10.00–123.50)	192.85 (40.00–3672)	1163.00 (36.11–4000)	317.7 (15.18–4000)
4 years after ^131^I therapy (i)	12.68 (10.00–56.86)	171.75 (41.30–1906)	1075.00 (29.30–4000)	709.40 (22.55–4000)
5 years after ^131^I therapy (j)	12.40 (10.00–39.00)	150.00 (45.10–1547)	1520.00 (130.80–4000)	_a
6 years after ^131^I therapy (k)	13.00 (10.00–39.00)	123.5 (50.30–1043)	1300 (290.00–1300)	_b

a, b: All patients in Group D reached the follow-up end <5 years after ^131^I therapy.

**Table:** 

TgAb (IU/ml)	A–B	A–C	A–D	B–C	B–D	C–D
	p-Values					
Preoperative	<0.001	0.052	<0.001	0.079	0.550	0.068
1 month after surgery	<0.001	0.003	<0.001	0.145	0.571	0.160
Before ^131^I therapy	<0.001	<0.001	<0.001	0.559	0.290	0.212
1 month after ^131^I therapy	<0.001	<0.001	<0.001	0.568	0.108	0.134
6 months after ^131^I therapy	<0.001	<0.001	<0.001	0.729	0.221	0.505
1 year after ^131^I therapy	<0.001	<0.001	<0.001	0.094	0.164	0.529
2 years after ^131^I therapy	<0.001	<0.001	<0.001	0.004	0.208	0.236
3 years after ^131^I therapy	<0.001	<0.001	<0.001	<0.001	0.482	0.060
4 years after ^131^I therapy	<0.001	<0.001	<0.001	<0.001	0.383	0.505
5 years after ^131^I therapy	<0.001	<0.001	_	0.004	_	_
6 years after ^131^I therapy	<0.001	0.004	_	0.03	_	_

**Table:** 

p-Values of the time point comparisons										
TgAb (IU/ml)	a–b	b–c	c–d	d–e	e–f	f–g	g–h	h–i	i–j	j–k
	p-Value									
Group A	<0.01	<0.01	0.235	<0.01	<0.01	<0.01	0.046	0.167	0.693	0.422
Group B	0.146	0.022	0.689	0.191	0.284	0.686	0.526	0.340	0.518	0.836
Group C	0.707	0.573	0.888	0.888	0.439	0.557	0.496	0.977	0.516	0.905
Group D	0.529	0.253	0.565	0.414	0.738	0.947	0.650	0.877	_	_

TgAb, antithyroglobulin antibody.

### Comparisons of the Variations in Antithyroglobulin Antibody Relative to the Preoperative Levels

At every time point of follow-up after surgery, variations in TgAb relative to the preoperative levels were calculated as [(TgAb at every time point) − (preoperative TgAb)]/(preoperative TgAb). The variations in TgAb in Group A were significantly larger than those in Groups B, C, and D at every time point of the follow-up except “1 month after surgery” (p < 0.001). The variations in TgAb in Group B were significantly larger than those in Group C at every time point except “1 month after surgery” (p < 0.05). The variations in TgAb in Group C were significantly larger than those in Group D after ^131^I therapy (p < 0.05) (**Table 3**).

**Table 3:** Comparison of the variations in TgAb relative to the preoperative levels.

Variation in TgAb relative to the preoperative level (%)	Tumour free (A)	Uncertain (B)	Incomplete biochemical response (C)		Structural disease (D)	
1 month after surgery	−34.74 (−92.93 to 3.99)	−22.27 (−74.51 to 90.49)	−7.46 (−73.50 to 75.26)		−18.90 (−66.03 to 39.76)	
Before ^131^I therapy	−76.34 (−97.13 to 2.63)	−49.71 (−90.51 to 14.67)	−19.33 (−87.27 to 57.50)		−43.69 (−94.65 to 63.98)	
6 months after ^131^I therapy	−89.14 (−98.39 to −18.95)	−65.11 (−94.29 to 112.54)	−9.16 (−85.85 to 117.01)		−50.74 (−98.62 to 670.64)	
1 year after ^131^I therapy	−93.39 (−99.27 to −31.79)	−67.53 (−96.72 to 293.96)	0 (−83.24 to 31.99)		−67.70 (−99.53 to 656.72)	
2 years after ^131^I therapy	−94.84 (−99.49 to −31.96)	−67.98 (−96.25 to 577.82)	7.34 (−82.60 to 214.31)		−72.73 (−99.58 to 656.72)	
**p-Values of comparisons of groups**						
**Variation in TgAb relative to the preoperative level**	**A–B**	**A–C**	**A–D**	**B–C**	**B–D**	**C–D**
	**p-Value**					
1 month after surgery	0.064	0.001	0.080	0.124	0.794	0.233
Before ^131^I therapy	<0.001	<0.001	<0.001	0.009	0.271	0.273
6 months after ^131^I therapy	<0.001	<0.001	<0.001	0.001	0.567	0.028
1 year after ^131^I therapy	<0.001	<0.001	<0.001	<0.001	0.487	0.011
2 years after ^131^I therapy	<0.001	<0.001	<0.001	<0.001	0.483	0.003

TgAb, antithyroglobulin antibody.

### Prediction Efficacy of Variations in Antithyroglobulin Antibody Relative to the Preoperative Levels on Clinical Outcomes

**Figure 3:**
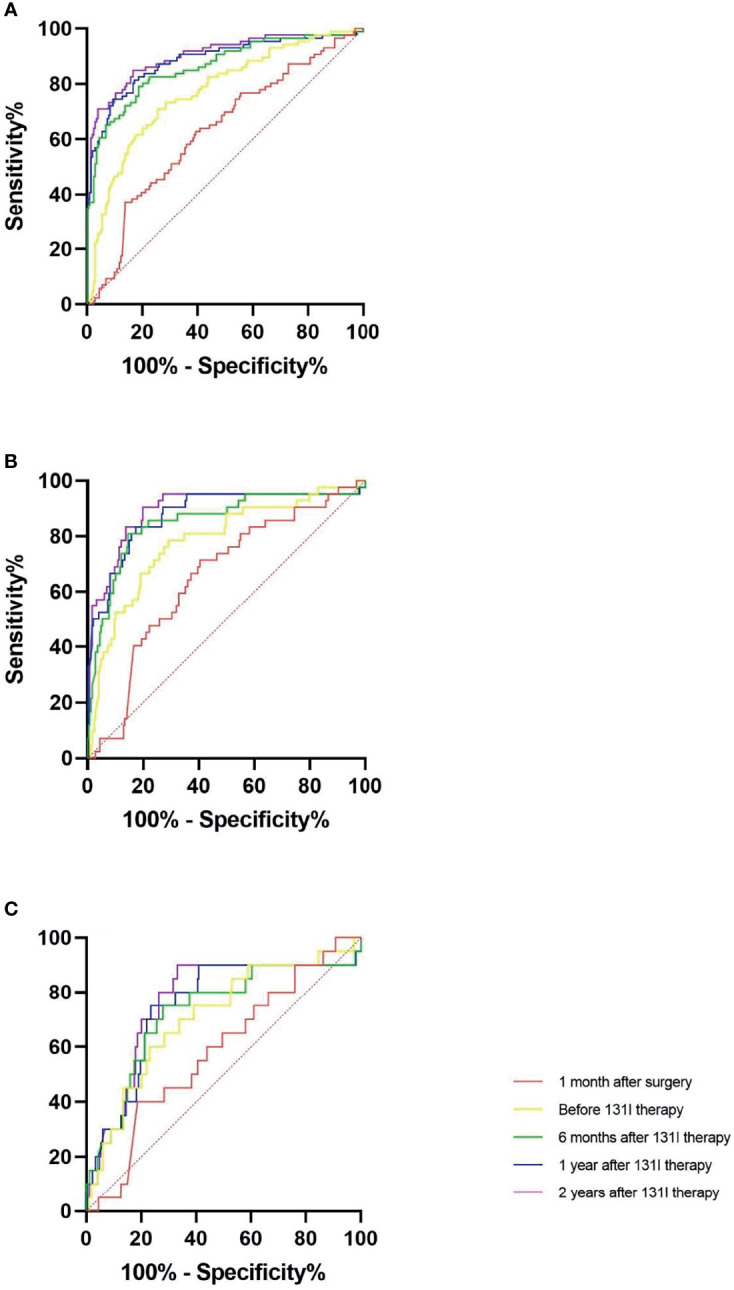
Diagnostic benefit of the variations in antithyroglobulin antibody (TgAb) relative to the preoperative levels in predicting the clinical outcome during receiver operating characteristic (ROC) analyses. **(A)** Tumour-free *versus* uncertain + incomplete biochemical response + structural disease at the end of follow-up. **(B)** Tumour free + uncertain *versus* incomplete biochemical response + structural disease at the end of follow-up. **(C)** Tumour-free + uncertain + incomplete biochemical response *versus* structural disease at the end of follow-up.

ROC analyses to predict the clinical outcomes at the end of follow-up were used in the following three comparisons: 1) tumour free with all others, 2) tumour free + uncertain group with incomplete biochemical response + structural disease group, and 3) the structural disease group with all other groups (**Figure 3** and **Table 4**).

**Table 4:** Diagnostic benefit of variations in TgAb relative to the preoperative levels in predicting the clinical outcome (by ROC analyses).

**Tumour free versus uncertain + incomplete biochemical response + structural disease at the end of follow-up.**								
**Variation in TgAb relative to the preoperative level**	**AUC**	**p-Value**	**Cut-off**	**Sens%**	**Spec%**	**PPV%**	**NPV%**	**DA%**
1 month after surgery	0.628	0.001	−24.2%	62.8	60.6	40.3	79.2	61.1
Before ^131^I therapy	0.771	<0.001	−63.4%	70.9	74.4	43.8	88.2	63.3
6 months after ^131^I therapy	0.862	<0.001	−77.9%	79.1	81.3	64.2	90.2	80.6
1 year after ^131^I therapy	0.884	<0.001	−77.4%	74.4	90.6	77.1	89.3	85.8
2 years after ^131^I therapy	0.901	<0.001	−88.6%	84.9	83.3	67.3	91.4	82.7
**Tumour free +uncertain versus incomplete biochemical response + structural disease at the end of follow-up.**								
**Variation in TgAb relative to the preoperative level**	**AUC**	**p-Value**	**Cut-off**	**Sens%**	**Spec%**	**PPV%**	**NPV%**	**DA%**
1 month after surgery	0.652	0.002	−22.9%	71.4	59.5	23.1	92.5	61.2
Before ^131^I therapy	0.782	<0.001	−60.4%	78.6	70.9	31.4	95.1	72.0
6 months after ^131^I therapy	0.853	<0.001	−65.5%	81.0	85.4	48.6	96.3	84.8
1 year after ^131^I therapy	0.877	<0.001	−75.4%	83.3	82.6	44.9	96.7	82.7
2 years after ^131^I therapy	0.894	<0.001	−83.8%	90.5	80.2	43,7	98.0	81.7
**Tumour free +uncertain versus incomplete biochemical response + structural disease at the end of follow-up.**								
**Variation in TgAb relative to the preoperative level**	**AUC**	**p-Value**	**Cut-off**	**Sens%**	**Spec%**	**PPV%**	**NPV%**	**DA%**
1 month after surgery	0.584	0.209	−22.9%	60.0	56.1	9.2	95.0	56.4
Before ^131^I therapy	0.710	0.002	−45.6%	60.0	77.0	12.7	94.5	74.7
6 months after ^131^I therapy	0.728	0.001	−73.7%	75.0	72.1	16.7	97.5	72.3
1 year after ^131^I therapy	0.750	<0.001	−75.4%	75.0	76.6	19.2	97.6	76.5
2 years after ^131^I therapy	0.764	<0.001	−88.6%	90.0	66.9	16.8	98.9	68.5

TgAb, antithyroglobulin antibody; ROC, receiver operating characteristic; AUC, area under the curve; PPV, positive predictive value; NPV, negative predictive value; DA, diagnostic accuracy.

The area under the curve (AUC) values measured during ROC analyses showed good diagnostic values, and the p-value was <0.001 in most cases. Considering the AUC values, the prediction efficacy of the variations in TgAb on the clinical outcome of tumour free was better than on other clinical outcomes, and the prediction efficacy of the variations in TgAb on the clinical outcome of tumour free at time points after ^131^I therapy was better than that before ^131^I therapy.

### Predictors of the Tumour-Free Status at the End of Follow-Up in Univariate and Multivariate Analyses

By applying univariate analyses, some risk factors were identified as predictors of tumour-free survival at the end of follow-up (**Table 5**). In multivariate analysis, variations in the TgAb at 1 year after ^131^I therapy ≥ −77.4%, and 2 years after ^131^I therapy ≥ −88.6% were independent predictive factors for a tumour-free status at the end of follow-up (**Table 6**).

**Table 5:** Univariate analysis of predictors of a tumour-free clinical outcome.

Characteristics	Disease free	Non-disease free	p
No. of patients	203	86	
Age at diagnosis (years), median (range)			0.791
<55	187 (92.12%)	80 (93.02%)	
≥55	16 (7.88%)	6 (6.98%)	
Sex			0.025
Male	24 (11.82%)	19 (22.09%)	
Female	179 (88.18%)	67 (77.91%)	
Surgery			0.006
TT + central neck lymph node dissection	143 (70.44%)	46 (53.49%)	
TT + central and lateral neck lymph node dissection	60 (29.56%)	40 (46.51%)	
Histology			0.79
Classic PTC	195 (96.06%)	84 (97.67%)	
FV-PTC	4 (1.97%)	1 (1.16%)	
DSV-PTC	4 (1.97%)	1 (1.16%)	
Primary tumour size (cm), median (range)	1.0 (0.3–6.0)	1.7 (0.5–6.0)	<0.001
No. of LN metastases, median (range)	3 (0–36)	6 (0–35)	<0.001
Bilateral tumour	73 (35.96%)	30 (34.88%)	0.861
Multifocal tumour	83 (40.89%)	35 (40.70%)	0.976
Extrathyroidal extension			<0.001
No invasion	47 (23.15%)	15 (17.44)	
Microscopic	150 (73.89%)	48 (55.81%)	
Macroscopic	6 (2.96%)	23 (26.74%)	
T			<0.001
T1	169 (83.25%)	41 (47.67%)	
T2	25 (12.32%)	14 (16.28%)	
T3	8 (3.94%)	18 (20.93%)	
T4	1 (0.49%)	13 (15.12%)	
N			<0.001
N0	34 (16.75%)	5 (5.81%)	
N1a	119 (58.62%)	41 (47.67%)	
N1b	50 (24.63%)	40 (46.51%)	
Staging			
<55 years			
I	187 (92.12%)	80 (93.02%)	
I	0 (0)	0 (0)	
≥55 years			<0.001
I	3 (1.48%)	0 (0)	
II	13 (6.40%)	1 (1.16%)	
III	0 (0)	3 (3.49%)	
IV	0 (0)	2 (2.33%)	
Initial risk stratification			<0.001
Low risk	14 (6.90%)	3 (3.49%)	
Intermediate risk	168 (82.76%)	54 (62.79%)	
High risk	21 (10.34%)	29 (33.72%)	
Cumulative ^131^I-administered activities (mCi)			0.337
30	17 (8.37%)	3 (3.49%)	
50	5 (2.46%)	3 (3.49%)	
100	171 (84.24%)	73 (84.88%)	
150	10 (4.93%)	7 (8.14%)	
Time interval between the first ^131^I treatments to surgery (months), median (range)	4.1 (1.2–12.0)	4.0 (0.8–12.0)	0.463
Preoperative serum biomarkers level			
TSH (μIU/ml), median (range)	3.08 (0.01–28.15)	2.99 (0.01–52.99)	0.388
FT3 (pmol/L), median (range)	4.85 (3.27–8.53)	4.80 (3.32–6.81)	0.526
FT4 (pmol/L), median (range)	16.25 (9.63–27.30)	16.34 (12.30–24.48)	0.723
TgAb (IU/ml), median (range)	320.60 (44.92–4000)	942.45 (44.74–4000)	<0.001

PTC, papillary thyroid cancer; FV, follicular variant; DSV, diffuse sclerosing variant; LN, lymph node; TSH, thyroid-stimulating hormone; TgAb, antithyroglobulin antibody.

**Table 6:** Multivariate analysis of predictors of a tumour-free clinical outcome.

Parameters	OR	95%CI	P
Sex			
Male	1		
Female	0.355	0.111–1.135	0.081
Surgery			
TT+ central neck lymph node dissection	1		
TT+ central and lateral neck lymph node dissection	0.677	0.139–3.288	0.628
Extrathyroidal extension			
No invasion	1		
Microscopic	1.703	0.097–29.976	0.716
Macroscopic	0.890	0.061–13.048	0.932
T			
T1	1		
T2	0.016	0.000–0.876	0.043
T3	0.060	0.001–3.207	0.166
T4	0.247	0.012–5.162	0.367
N			
N0	1		
N1a	0.820	0.100–6.732	0.854
N1b	1.120	0.212–5.925	0.894
Initial risk stratification			
Low risk	1		
Intermediate risk	0.283	0.025–3.234	0.310
High risk	0.461	0.124–1.712	0.247
The variation of TgAb relative to preoperative level			
Before ^131^I therapy <−63.4%	1		
Before ^131^I therapy ≥−63.4%	0.600	0.193–1.865	0.377
6 months after ^131^I therapy <−77.9%	1		
6 months after ^131^I therapy ≥−77.9%	3.098	0.975–9.845	0.055
1 year after ^131^I therapy <−77.4%	1		
1 year after ^131^I therapy ≥−77.4%	4.875	1.651–14.395	0.004
2 years after ^131^I therapy <−88.6%	1		
2 years after ^131^I therapy ≥−88.6%	9.919	3.185–30.885	<0.001

OR, odds ratio; TT, total thyroidectomy; TgAb, antithyroglobulin antibody.

### Integrating the Variation in Antithyroglobulin Antibody With American Thyroid Association Risk Categories

In our study cohort, when patients were stratified according to the ATA categories, the disease-free status declined progressively, with the level of initial risk estimated at 82.35% in the low-risk patients, 75.68% in the intermediate-risk patients, and 42.0% in the high-risk patients.

When the variation of TgAb ≥ −77.9% at 6 months after ^131^I therapy was incorporated as a variable in the ATA categories, both intermediate- and high-risk patients showed a significantly increased chance of being tumour free (from 75.68% to 91.28% and 42.0% to 78.26%, respectively). Similarly, when the variation of TgAb ≥ −88.6% at 2 years after ^131^I therapy was incorporated as a variable in the ATA categories, both intermediate- and high-risk patients also showed a significantly increased chance of being tumour free (from 75.68% to 93.88% and 42.0% to 82.61%, respectively) (**Table 7**).

**Table 7:** Restratification of the ATA risk categories according to the variation in TgAb relative to the preoperative level.

Disease free	ATA initial risk of recurrence classification (n = 289)		
	Low (n = 17)	Intermediate (n = 222)	High (n = 50)
	82.35% (14/17)	75.68% 168/222)	42.0% (21/50)
Variation in TgAb at 6 months after ^131^I therapy			
≥−77.9% (n = 184)	91.67% (11/12)	91.28% (136/149)	78.26% (18/23)
<−77.9% (n = 105)	60% (3/5)	43.84% (32/73)	11.11% (3/27)
p	0.474	<0.001	0.004
Variation in TgAb at 2 years after ^131^I therapy			
≥−88.6% (n = 182)	100% (12/12)	93.88% (138/147)	82.61% (19/23)
<−88.6% (n = 107)	40% (2/5)	40% (30/75)	7.41% (2/27)
p	0.246	<0.001	0.001

ATA, American Thyroid Association; TgAb, antithyroglobulin antibody.

## Discussion

Usually, TgAb is a serum biomarker for the diagnosis and follow-up of thyroid autoimmune diseases. However, because of the following three characteristics, TgAb has been more valued as a prognostic indicator for DTC patients: 1) approximately 20%~30% of patients with DTC are TgAb positive on initial postoperative assessment (14, 15); 2) the presence of TgAb compromises the authenticity of Tg, and it is recommended to measure the level of TgAb during Tg measurement; 3) ideally, for DTC patients who undergo TT and ^131^I therapy, the TgAb level should decrease until undetectable without stimulation of Tg. Many cross-sectional studies focus on the relationship between the level of TgAb and DTC, but the prognostic significance of the TgAb status (positive/negative) is unclear (16). Compared with observing TgAb at a single time point, TgAb trends appear to have more clinical usefulness as a surrogate tumour marker in the surveillance of TgAb-positive DTC patients (16) because the trend of TgAb is associated with higher tumour metabolism (17). Most existing studies have concluded that an increase in TgAb can indicate disease presence (18–20), and some studies empirically used a reduction of 50% of TgAb to group subjects, finding that a reduction >50% can represent a good prognosis (21). Based on these studies, our study described overall TgAb change trends after surgery in preoperative TgAb-positive DTC patients and compared changes in TgAb among patients with different clinical outcomes, aiming to further elucidate the prognostic value of TgAb.

The current study demonstrated the prognostic value of variations in TgAb relative to the preoperative level after surgery in predicting clinical outcomes. In particular, variations of TgAb at 6 months after ^131^I therapy ≥ −77.9% and at 2 years after ^131^I therapy ≥ −88.6% were excellent predictors of tumour-free survival, with negative predictive values (NPVs) as high as 90.2% and 91.4%, respectively. Our study indicated that the integration of the variations of TgAb into the ATA categories more effectively predicted the clinical outcome of tumour-free patients. Additionally, 23.65% of DTC patients had positive preoperative TgAb, which was comparable with the 23.2% reported by Lee et al. (22). Using the change in TgAb during follow-up, we found that TgAb in the tumour-free group declined significantly after surgery and ^131^I therapy, but we did not observe this phenomenon in the other three groups. Additionally, after 1–2 years of ^131^I therapy, the TgAb of patients in the tumour-free group stabilised at a very low level. However, in the uncertain group, the TgAb decline was slow. In the incomplete biochemical response group, TgAb increased. In the structural disease group, the TgAb levels continued to be high and positive. This result was consistent with the general view that persistent/increasing trends in TgAb were associated with a compromised DTC prognosis (16, 23). Additionally, we observed a slight rise in TgAb at 1 month after ^131^I therapy in most groups, likely attributed to the response to an acute increase in Tg antigen after ^131^I therapy (24).

In this study, to predict the clinical outcomes, five variations in TgAb—1 month after surgery, before ^131^I therapy, and 6 months, 1 year, and 2 years after ^131^I therapy—were evaluated in 289 preoperative TgAb-positive DTC patients. The AUC values obtained in the ROC analysis were mostly good (>0.75); in one-third of the cases, they were excellent (>0.85). The variation of TgAb > −88.6% at 2 years after ^131^I therapy showed the highest AUC value (0.901) to predict a tumour-free status, with 84.9% sensitivity, 83.3% specificity, 82.7% diagnostic accuracy, 91.4% NPV, and 67.3% positive predictive value (PPV). For the good performance of the variation of TgAb in predicting a disease-free status, we combined the three groups with the tumour-free group into the non-disease-free group, and we found that the variations of TgAb > −77.4% at 1 year after ^131^I therapy, and TgAb > −88.6% at 2 years after ^131^I therapy were independent predictive factors for a tumour-free status at the end of follow-up. We also combined the subgroups because both the uncertain clinical outcome and incomplete biochemical response reflect that residual cancer could not be ruled out.

The prognostic role of TgAb has been studied in some studies (11, 12, 15, 25). Although the studies differed in patient grouping, the authors found that the trend and reduction of TgAb > 50% are critical prognostic factors. In our study, the cut-off values of TgAb reduction at different time points in the follow-up were determined by ROC analysis and were generally consistent with other studies. The major advantage of our study is that a relatively large population (considering that TgAb-positive patients account for only 20%~30% of DTC patients) undergoing standardised diagnostic and therapeutic processes in West China Hospital (third-class hospital) could be evaluated. Additionally, a strength of our study is that the changes of TgAb during follow-up are shown intuitively, providing new insight into the prognostic value of TgAb in the clinical outcomes and integrating the variation of TgAb into the ATA risk categories to increase the chance of having a tumour-free status in intermediate- and high-risk patients.

However, our study has limitations. First, this study has a single-centre and retrospective study design, which may lead to selection biases. Second, although a median follow-up period of 4.83 years was a reasonable starting point to assess the clinical outcome, a longer follow-up is still necessary to assess the risk of late recurrences. However, most recurrences occurred within the first 5 years of follow-up after initial therapy (26). Finally, although we concluded that the variation of TgAb had good prognostic efficacy in disease-free patients, the prognostic efficacy was poor to predict disease persistence/recurrence. Under this circumstance, more imaging examinations, such as ^131^I whole body scan (WBS) and FDG PET/CT, may be needed to identify whether the disease has recurred.

## Conclusion

For preoperative TgAb-positive DTC patients, the variations of TgAb > −77.9% at 6 months after ^131^I therapy and TgAb > −88.6% at 2 years after ^131^I therapy showed good prognostic efficacy for a tumour-free status. Their incorporation as variables in the ATA risk stratification system could more accurately predict disease-free survival.

## Data Availability Statement

The original contributions presented in the study are included in the article/supplementary material. Further inquiries can be directed to the corresponding author.

## Ethics Statement

Our research was approved by the Ethics Committee of West China Hospital of Sichuan University.

## Author Contributions

All the authors contributed to the study conception and design. Material preparation, data collection and analysis, writing-original draft, and writing-review and editing were performed by QL. Data collection and analysis were performed by MY. Writing-review and editing was performed by GL. And all the authors commented on previous versions of the manuscript. All authors contributed to the article and approved the submitted version.

## Conflict of Interest

The authors declare that the research was conducted in the absence of any commercial or financial relationships that could be construed as a potential conflict of interest.

## Publisher’s Note

All claims expressed in this article are solely those of the authors and do not necessarily represent those of their affiliated organizations, or those of the publisher, the editors and the reviewers. Any product that may be evaluated in this article, or claim that may be made by its manufacturer, is not guaranteed or endorsed by the publisher.
