# Corrigendum: Antithyroglobulin Antibody Variation During Follow-Up Has a Good Prognostic Value for Preoperative Antithyroglobulin Antibody-Positive Differentiated Thyroid Cancer Patients: A Retrospective Study in Southwest China

**DOI:** 10.3389/fendo.2022.899184

**Published:** 2022-04-27

**Authors:** Qianhui Liu, Mengting Yin, Guixing Li

**Affiliations:** Department of Laboratory Medicine, West China Hospital of Sichuan University, Chengdu, China

**Keywords:** thyroid cancer, prognosis, antithyroglobulin, biomarker, DTC (differentiated thyroid cancer)

In the original article, there was a mistake in **Table 6** as published. We recently found by ourselves that the p values of T2 and “6 months after ^131^I therapy ≥ − 77.9%” were incorrect owing to the careless recording. Furthermore, the corresponding text of **Table 6** should be corrected as “*In multivariate analysis, variations in the TgAb at 1 year after ^131^I therapy ≥ − 77.4% and 2 years after ^131^I therapy ≥-88.6% were independent predictive factors for a tumour-free status at the end of follow-up.*” The corrected **Table 6** appears below.

**Table 6:** Multivariate analysis of predictors of a tumour-free clinical outcome.

Parameters	OR	95%CI	P
Sex			
Male	1		
Female	0.355	0.111–1.135	0.081
Surgery			
TT+ central neck lymph node dissection	1		
TT+ central and lateral neck lymph node dissection	0.677	0.139–3.288	0.628
Extrathyroidal extension			
No invasion	1		
Microscopic	1.703	0.097–29.976	0.716
Macroscopic	0.890	0.061–13.048	0.932
T			
T1	1		
T2	0.016	0.000–0.876	0.043
T3	0.060	0.001–3.207	0.166
T4	0.247	0.012–5.162	0.367
N			
N0	1		
N1a	0.820	0.100–6.732	0.854
N1b	1.120	0.212–5.925	0.894
Initial risk stratification			
Low risk	1		
Intermediate risk	0.283	0.025–3.234	0.310
High risk	0.461	0.124–1.712	0.247
The variation of TgAb relative to preoperative level			
Before ^131^I therapy <−63.4%	1		
Before ^131^I therapy ≥−63.4%	0.600	0.193–1.865	0.377
6 months after ^131^I therapy <−77.9%	1		
6 months after ^131^I therapy ≥−77.9%	3.098	0.975–9.845	0.055
1 year after ^131^I therapy <−77.4%	1		
1 year after ^131^I therapy ≥−77.4%	4.875	1.651–14.395	0.004
2 years after ^131^I therapy <−88.6%	1		
2 years after ^131^I therapy ≥−88.6%	9.919	3.185–30.885	<0.001

OR, odds ratio; TT, total thyroidectomy; TgAb, antithyroglobulin antibody.

In the original article, there was an expression error. A correction has been made to the **Results** section ”Change Trends of Antithyroglobulin Antibody and Comparisons of the Levels of Antithyroglobulin Antibody at Different Time Points”: “*In all groups except Group C, small increases were observed in the levels of TgAb at 1 month after ^131^I therapy (d) compared with those before ^131^I therapy (c)*”.

Moreover, in the original article, there were two errors in Discussion. A correction has been made to **Discussion**, paragraph 2, the last sentence: “*Additionally, we observed a slight rise in TgAb at 1 month after ^131^I therapy in most groups*”, and **Discussion**, paragraph 3: “*and we found that the variations of TgAb > −77.4% at 1 year after ^131^I therapy, and TgAb > −88.6% at 2 years after ^131^I therapy were independent predictive factors for a tumour-free status at the end of follow-up.*”

The authors apologize for these errors and state that these do not change the scientific conclusions of the article in any way. The original article has been updated.

## Publisher’s Note

All claims expressed in this article are solely those of the authors and do not necessarily represent those of their affiliated organizations, or those of the publisher, the editors and the reviewers. Any product that may be evaluated in this article, or claim that may be made by its manufacturer, is not guaranteed or endorsed by the publisher.

